# Editorial: Clinical Evaluation Criteria for Aging and Aging-Related Multimorbidity

**DOI:** 10.3389/fgene.2021.764874

**Published:** 2021-09-14

**Authors:** Ilia Stambler, Alexey Moskalev

**Affiliations:** ^1^ Vetek (Seniority) Association–The Movement for Longevity and Quality of Life, Tel Aviv, Israel; ^2^ International Longevity Alliance (ILA), Paris, France; ^3^ Institute of Biology, Komi Science Center of Russian Academy of Sciences, Syktyvkar, Russia; ^4^ Russian Clinical and Research Center of Gerontology, Moscow, Russia

**Keywords:** aging, longevity, multimorbidity, biomarkers, clinical endpoints, functional evaluation, regulation, clinical trials

It is becoming increasingly clear that population aging brings a train of degenerative, malignant and other chronic diseases, such as cancer, type 2 diabetes, chronic obstructive pulmonary disease, neurodegenerative diseases, heart disease, aggravation of infectious diseases. This is also accompanied by other diverse functional, physical and mental impairments. These conditions do not emerge separately from each other, but have related aetiologies and mutually exacerbate each other. This multitude of morbid conditions has been often termed as “multimorbidity” or “co-morbidity.” Moreover, it has been suggested that a promising approach to address the entire host of old-age-related morbidities would be by treating their underlying determinative factors–namely fundamental degenerative processes of aging.

Yet, there is currently no agreed method to estimate the direct effects of therapy on tackling the aging process as such, for which there is presently no agreed formal or clinical definition or criteria. Moreover, essentially, there is no agreed formal or clinical definition and criteria for old-age multimorbidity either. Correspondingly, there are no agreed scientifically grounded criteria to select interventions against degenerative aging and old-age multimorbidity or to evaluate their effectiveness. There are clinical methods to diagnose individual age-related diseases and dysfunctions, and assess interventions against those individual diseases and dysfunctions. Yet their integrated evaluation as “aging-related ill health” or “multimorbidity,” as well as the selection and evaluation of effective interventions against these conditions, remain as unresolved methodological challenges. As a result, there is no agreed formal conceptual basis for incentivizing industrial development, nor regulatory adoption, of diagnostics and therapies against degenerative aging and aging-related multimorbidity (Moskalev et al., 2016; Stambler, 2017).

The main aim of the current Research Topic was to contribute to establishing the methodological basis for developing, and regulatory adoption of, diagnostic criteria for aging and aging-related multimorbidity. The articles published in this research topic provided a broad and diverse exploration of clinical criteria for aging and old-age multimorbidity that utilized diverse physiological, functional, genetic, epigenetic and other biomarkers and methods of bioinformatics. Of special interest were the selecting of the most informative and economic diagnostic parameters (biomarkers and functional essays) for aging and old-age multimorbidity and developing guidelines and analytical methodologies for clinical testing of interventions against degenerative aging and old-age multimorbidity.

The articles published in this research topic reconfirmed the multiplicity of approaches, and demonstrated once again how far we are yet from any kind of general or “consensus” agreement about the metrics of aging and aging-related multimorbidity. The combinations of examined parameters were quite distinct for all the researchers, according to their diverse methodological backgrounds, the measurement techniques and the data that were convenient, available and affordable. Practically, there were as many methodological focuses of evaluation as there were studies. This diversity is a salient characteristic of the broad and complex aging field, and is likely unavoidable, but it showcased once again that in the clinical evaluation of aging and multimorbidity, there may be a common language, but not many common rules. Yet, the articles of this research topic outlined some of the potential directions for more integration, harmonization and standardization of the aging evaluation field.

First of all, they emphasized the need for a systemic composite approach that would combine multiple evaluation criteria and parameters from multiple organ systems (the nervous system and cognition, respiratory and cardiovascular system, muscular system, etc.) and from different levels of biological organization. Thus, in this topic, Vaiserman and Krasnienkov, in their review of telomere length as a marker of biological age, emphasized the importance of combined assessment of biomarkers of aging. Though telomere length has been widely used as a purported biomarker of aging, the authors argued that as a stand alone measure, it may have limited predictive value and clinical importance, yet in combination with other parameters (such as certain immune parameters, indices of epigenetic age, indices of homeostatic dysregulation, frailty index, etc.) it can improve the risk evaluation for aging-related ill health (Vaiserman and Krasnienkov). Krut’ko et al. reporting their method and computer system for dialog optimization of aging biomarker panels for biological age assessment, reconciled with a possibly unlimited multiplicity of approaches to aging evaluation, and argued for the need to select and optimize particular panels of biomarkers for particular tasks, using pre-defined optimization criteria (Krut’ko et al.). In this topic, Li et al., in their study of hamartin as an endogenous neuroprotective molecule induced by hypoxic preconditioning, they showcased that a fruitful approach to developing and selecting aging evaluation criteria may be by actual trials of potential geroprotecitive interventions (Li et al.).

Considering aging evaluation in diverse organ systems, Strasser stressed the clinical significance of assessing muscular fitness in secondary care, striving to improve practical guidelines for such assessment, with specific reference to older persons (Strasser). Gustafsson and Ulfhake provided an in depth review of the loss of muscle function and mass (sarcopenia) in the framework of human aging, healthspan and lifespan, with a special consideration of a potential neurogenic origin of sarcopenia, and argued for enhancing physical activity with appropriate predictive clinical monitoring (Gustafsson and Ulfhake).

Further emphasizing the interrelatedness of aging evaluation criteria, in this topic, the studies of Papathanasiou et al. and Chadjikyprianou et al. exemplified the types of methodological instruments commonly found in the toolkits of geriatric assessment and geriatric treatment. These chiefly relied on functional frailty assessments, especially cognitive assessments and self reports, with the aim to evaluate the relationships between multiple aspects of functional aging impairment. Thus, Papathanasiou et al. related between multimorbidity, trauma exposure, and frailty of older adults in the community (Papathanasiou et al.); while Chadjikyprianou et al. in their longitudinal neurocognitive study of aging, considered the relation of sex, age, education and APOE-4 with cognitive performance, utilizing such measures as executive functions and verbal episodic memory (Chadjikyprianou et al.).

The two last studies of this research topic provided further general directions toward harmonization of discourse on aging evaluation. Thus, Kim et al. suggested a compendium of age-related diseases and traits within a framework of genome-wide association studies (GWAS) and phenome-wide association studies (PheWAS). Even though the terms “age-related diseases (ARDs)” and “age-related traits (ARTs)” are commonly used, there are currently no accepted criteria for their definition, selection and registration. The authors make a step toward establishing an evidence-based list of such age-related diseases and traits, based on their prevalence with increasing age, suggesting a basis for further discussion and consensus building (Kim et al.). Finally, Hartmann et al. in their ranking of biomarkers of aging by citation profiling and effort scoring, provided an overview of different biomarker types often considered for aging assessment (routine and research laboratory biomarkers, physical capability and organ function parameters) and piloted their ranking system based on the biomarkers’ citation profile (the review count score) and estimated effort of use (effort score) (Hartmann et al.). Clearly, given the vast multitude of potential aging biomarkers and evaluation parameters, it is important to establish some sort of scoring or prioritization criteria, to facilitate their clinical use.

Altogether, this research topic showcased a wide variety of approaches and directions toward clinical evaluation criteria for aging and aging-related multimorbidity, and we hope it will contribute to stimulating the discussion and involvement for the further development of such evaluation criteria, which we believe are vitally important for healthy longevity research, development, application and education.
